# Establishment and evaluation of an indirect ELISA for detection of antibodies to goat *Klebsiella pneumonia*

**DOI:** 10.1186/s12917-021-02820-1

**Published:** 2021-03-05

**Authors:** Ruichang Chen, Hongqi Shang, Xiangyun Niu, Jin Huang, Yongqiang Miao, Zhou Sha, Liting Qin, He Huang, Duo Peng, Ruiliang Zhu

**Affiliations:** 1 Shandong Provincial Key Laboratory of Animal Biotechnology and Disease Control and Prevention, Shandong Agricultural University, Taian, China; 2 Shandong Provincial Engineering Technology Research Center of Animal Disease Control and Prevention, Shandong Agricultural University, Taian, China; 3 Shandong Newhope Liuhe Group Co., Ltd., Qingdao, China; 4 Department of Immunology and Infectious Diseases, Harvard T.H. Chan School of Public Health, Boston, MA 02115 USA

**Keywords:** Goat *Klebsiella pneumonia*, Polyclonal antibodies, Indirect ELISA detection method, Optimal working conditions, Clinical veterinary application

## Abstract

**Background:**

*Klebsiella pneumonia*, a Gram-negative bacterium belonging to the genus *Enterobacter*, causes many human and livestock diseases. Notably, infected goats may develop pneumonia, septicemia, which can lead to occasional death, resulting in great economic losses in goat-farming industry. However, there are little systematic methods for detection of goat *Klebsiella pneumoniae* in livestock production.

**Results:**

In this study, we developed a *Klebsiella pneumoniae* goat polyclonal antibody and established an indirect ELISA method to detect the *Klebsiella pneumoniae*. After screening and optimizing the conditions for detection, we determined the optimal working dilutions of the coated-bacterial antigen, the polyclonal antibody, and the enzyme-labeled secondary antibody that were 1:800 (2.99 × 10^7^ CFU/ml), 1:6400, and 1:5000, respectively. The optimal condition of coating and blocking were both 4 °C for 12 h. The optimal dilution buffers of bacterial antigen, the antibodies, and the blocking buffer were 0.05 mol/L carbonate buffer, 1% BSA phosphate buffer, and 1.5% BSA carbonate buffer, respectively. The cut-off value was determined to be 0.28, and the analytical sensitivity was 1:800 (dilution of a positive sample). Furthermore, there was no cross-reaction between the coated antigen and goat serum positive for antibodies against other bacteria, indicating that indirect ELISA could detect *Klebsiella pneumoniae* specifically in most cases. The average coefficients of variation of intra-assay and inter-assay were 4.37 and 5.17% indicating favorable reproducibility of indirect ELISA. In the detection of clinical veterinary samples, the positive rate of indirect ELISA was 6.74%, higher than that of conventional agglutination assays.

**Conclusions:**

Taken together, we successfully established an indirect ELISA method for detecting antibodies against *Klebsiella pneumoniae* in goats, which can be applied in production.

## Background

*Klebsiella pneumoniae* is an opportunistic pathogen parasitizing on the respiratory or intestinal tract of human and animals, and probably causes zoonotic disease such as meningitis, pneumonia, urinary tract inflammation, and even sepsis in the clinical veterinary, contributing to enormous potential threats to both human health and livestock production [1]. In China, along with increasing demand of goat livestock, goat husbandry industry has gradually adopted high-density breeding and fast fattening using concentrated feeding pattern [2]. However, in this process, overuse and even abuse of antibiotics contribute to an increase in the number of outbreaks of *Klebsiella pneumoniae* [3]. Seriously, *Klebsiella pneumoniae*-caused diseases and other secondary pathogenic bacteria infections eventually result in the death of goats [4]. Thus, an efficient, sensitive, and robust *Klebsiella pneumoniae* antibody detection method is highly desired to guide the prevention, intervention and control of the spread of *Klebsiella pneumoniae* in goat-farming industry [5].

Bacterial detection generally depends on phenotype or genotype [6]. Conventional phenotype-based laboratory techniques for the diagnosis of *Klebsiella pneumoniae* include pure culture, microscopic determination, biochemical examination are labor-intensive, time-consuming and easily interferred by other bacteria, even though regarded as the gold standards [7]. By contrast, genotype-based techniques are rapid and highly sensitive. Previous studies have established polymerase chain reaction (PCR)-based assays for human and fur-bearing animal (mink, raccoon dog, fox) *Klebsiella pneumoniae.* PCR technique variants, such as multiplex PCR and real-time PCR methods, are primary diagnostic tools in hospitals and laboratories [8]. There are also novel methods for the detection of *Klebsiella pneumoniae*, which are demonstrated to be more accurate and sensitive, like automatic bacteria identification system and mass spectrometry [9]. However, these methods could not be conducted on-site and in low resource-setting areas that lack precise equipment and trained personnel [10]. Furthermore, studies on goat-source *Klebsiella pneumoniae* are generally focused on isolation, identification, and epidemiological investigation while few studies on developing detection methods. Serological assay is also one of the commonly used detection methods in veterinary medicine [11]. In addition to phenotypic and genotypic identification of goat source *Klebsiella pneumoniae*, agglutination assay is occasionally used for antibody detection, but it has a main disadvantage of poor sensitivity. Indirect enzyme-linked immunosorbent assay (ELISA), one of the serological methods, is efficient, sensitive, specific, easy to operate, and can be scaled-up to apply to a large number of serum samples [12].

In line with this, we obtained a *Klebsiella pneumoniae* goat polyclonal antibody and established an indirect ELISA method. Furthermore, a total of 1320 serum samples from clinics in Shandong Province were subjected to assess whether our assay is feasible to deploy in clinical veterinary settings. Therefore, the developed an indirect ELISA detection assay is a rapid diagnostic method that could be applied to clinical veterinary diagnosis and epidemiological investigation of goat-sourced *Klebsiella pneumonia.*

## Results

### Preparation and identification of the *Klebsiella pneumoniae* goat polyclonal antibody

**Fig. 1:**
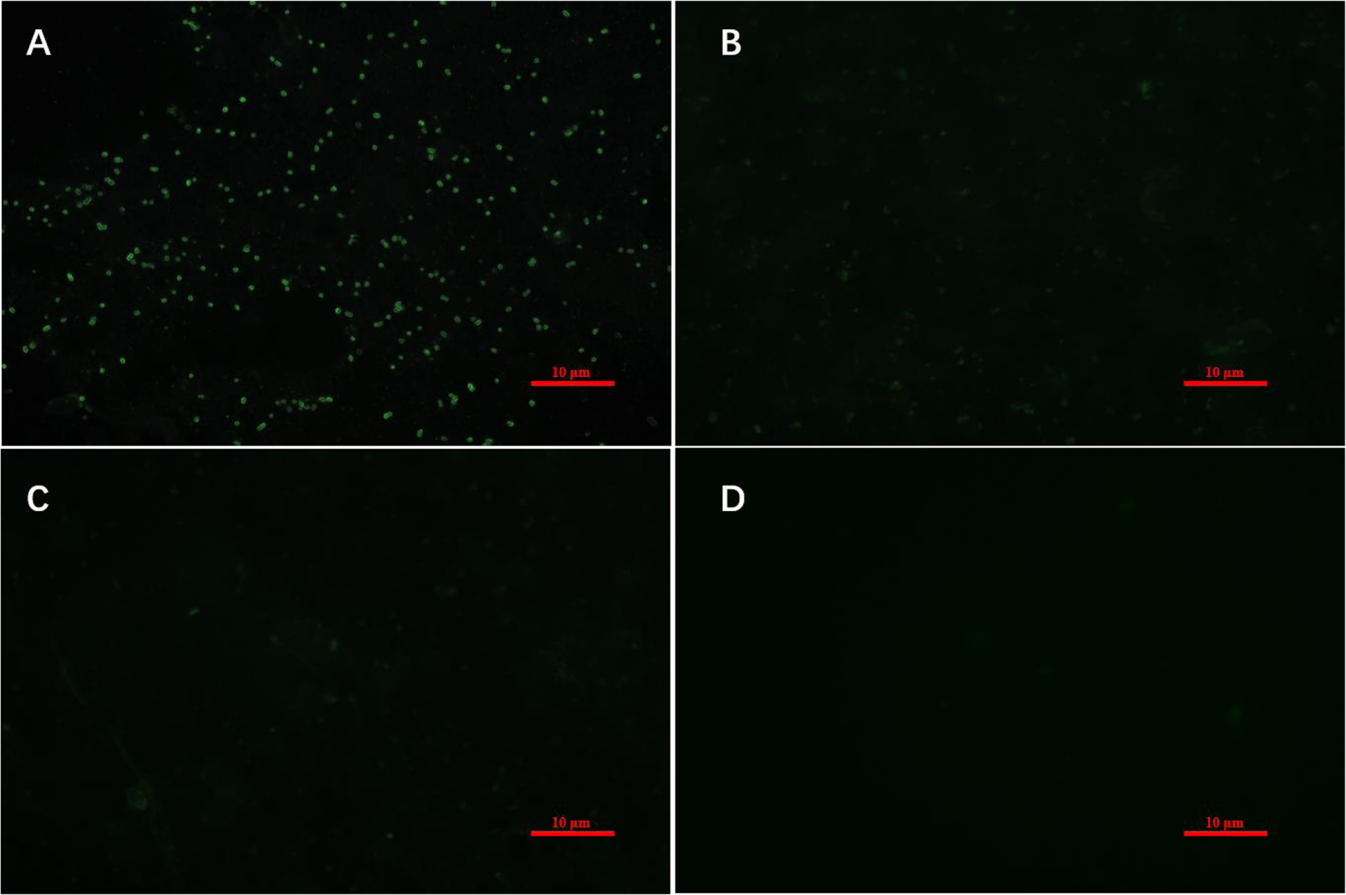
Indirect immunofluorescence verification of *Klebsiella pneumoniae* goat polyclonal antibody (1000 ×). Immunofluorescence was performed to detect the specificity of *Klebsiella pneumoniae* goat polyclonal antibody. A culture containing *Klebsiella pneumonia* was smeared on the slide and then stained using the *Klebsiella pneumoniae* goat polyclonal antibody as the primary antibody and FITC-labeled goat anti-rabbit IgG as the secondary antibody (**a**). Smears of *Escherichia coli* (**b**) and *Pasteurella* (**c**) were immunofluorescent stained by using the same polyclonal antibody. Smear without bacteria served as the negative control (**d**)

Using the agglutination test, we determined the agglutination value of the *Klebsiella pneumoniae* goat polyclonal antibody to be 9 log2. To identify the specificity of the *Klebsiella pneumoniae* goat polyclonal antibody, an indirect immunofluorescence assay was performed and there had been clear fluorescence on the *Klebsiella pneumoniae* smear by using the prepared polyclonal antibody (Fig. 1a). However, the smears of goat *Escherichia coli* and *Pasteurella* showed weak fluorescence (Fig. 1b and c), while the negative control group showed no fluorescence (Fig. 1d). Therefore, our results demonstrated that the *Klebsiella pneumoniae* goat polyclonal antibody can be used as the positive control serum for the detection of *Klebsiella pneumoniae*.

### Optimization of ELISA indirect conditions

**Fig. 2:**
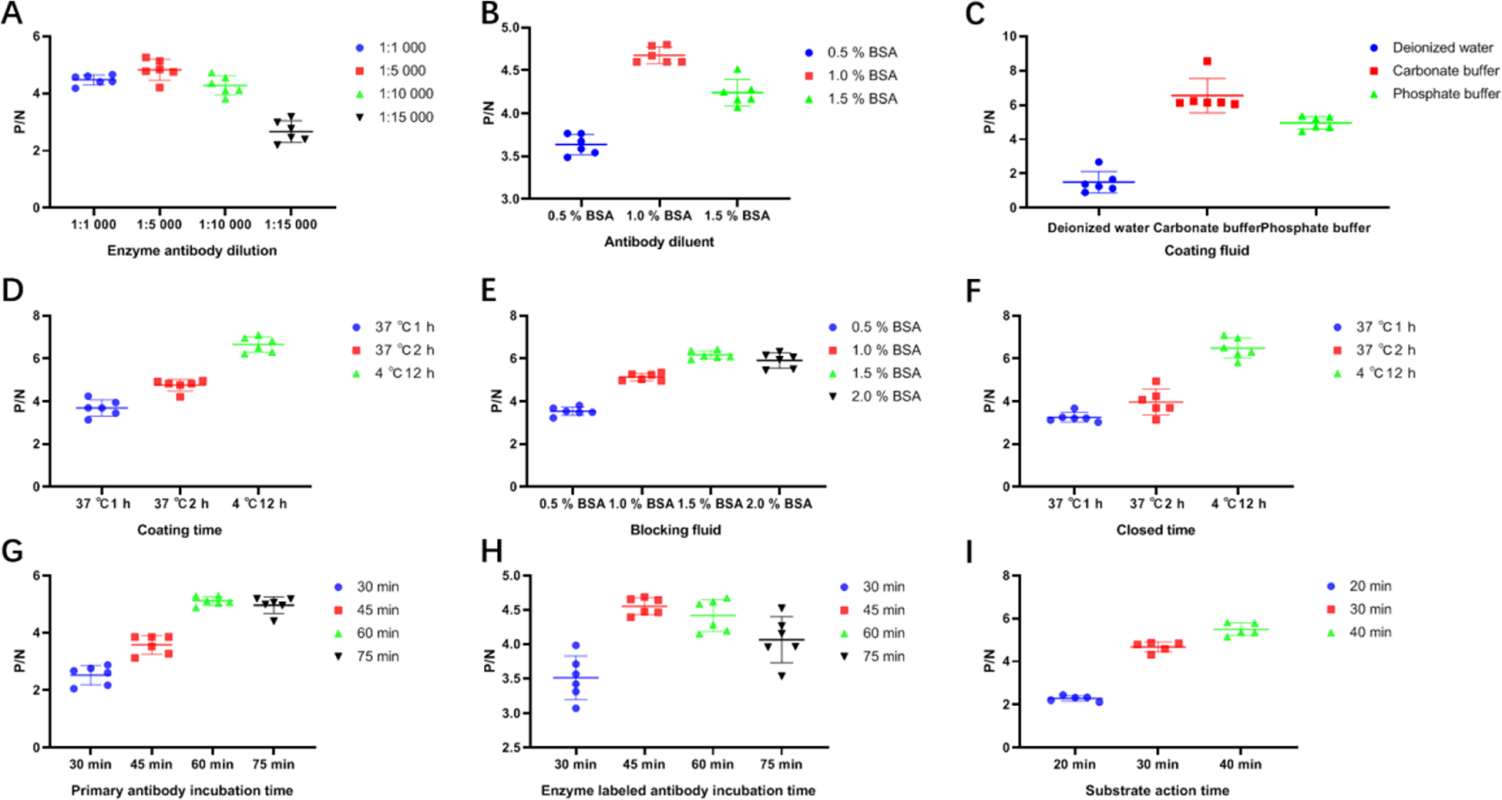
Optimization of ELISA indirect conditions. The optimal dilution of the HRP-conjugated Affinipure Rabbit Anti-Goat IgG was selected according to the P/N value (**a**), the antibody buffer (**b**), coating-buffer (**c**), coating-time /temperature (**d**), blocking-buffer (**e**), blocking-time/temperature (**f**), incubation time of the polyclonal antibody (**g**) and the HRP-conjugated IgG (**h**), and chromogenic time (**i**) were respectively determined in turn

To search for the optimal reaction condition, we performed a square matrix titration test (Table 1). The reaction conditions corresponding to the highest P/N values are generally considered to be the optimal conditions. As shown in Table 1, the optimal coating antigen dilution was determined to be 1:800, the corresponding antigen concentration is 2.99 × 10^7^ CFU/mL, and the optimal working dilution of polyclonal antibody is 1: 6400. The other reaction conditions were optimized by varying a single parameter at a time. The optimum dilution of HRP-conjugated Affinipure Rabbit Anti-Goat IgG was 1: 5000 (Fig. 2a). The optimal buffer for diluting both the polyclonal antibody and HRP-conjugated IgG was 1% BSA phosphate buffer (Fig. 2b). The antigen was diluted in 0.05 mol/L carbonate buffer at 4 °C for 12 h during the coating process (Fig. 2c and d). The best blocking solution was determined to be carbonate buffer containing 1.5% BSA and the plates were optimally blocked at 4 °C for 12 h (Fig. 2e and f). The polyclonal antibody and the HRP-conjugated Affinipure Rabbit Anti-Goat IgG were incubated for 60 min and 45 min, respectively (Fig. 2f and h). Furthermore, as the P/N value of the negative control is higher than 2.1 when the substrate was incubated for 40 min, the optimal reaction time was thereby determined to be 30 min (Fig. 2i, Table 2).

**Table 1:** P/N values of antigen- antibody indirect ELISA square matrix titration test

	*Klebsiella pneumoniae* goat polyclonal antibody dilutions										
Antigen dilution	1:1.0 × 10^2^	1:2.0 × 10^2^	1:4.0 × 10^2^	1:8.0 × 10^2^	1:1.6 × 10^3^	1:3.2 × 10^3^	1:6.4 × 10^3^	1:1.^28^ × 10^4^	1:2.56 × 10^4^	1:5.12 × 10^4^	1:1.024 × 10^4^
1:1 × 10^2^	2.119	3.036	3.063	3.165	4.456	4.760	5.061	4.700	4.565	3.272	2.311
1:2 × 10^2^	2.007	1.770	2.992	3.274	4.253	4.638	5.027	4.089	3.203	2.491	1.699
1:4 × 10^2^	1.744	2.384	2.852	3.399	4.430	4.955	4.624	3.763	2.899	2.412	1.951
1:8 × 10^2^	1.631	2.263	2.729	3.198	4.081	4.989	**5.122**	4.421	3.449	2.281	1.699
1:1.6 × 10^3^	1.629	1.881	2.719	3.388	3.919	4.243	4.677	4.705	3.341	2.342	1.699
1:3.2 × 10^3^	1.487	1.716	2.124	2.607	3.522	3.302	3.586	3.442	3.290	2.579	1.932
1:6.4 × 10^3^	0.808	1.670	1.979	2.407	3.393	3.260	3.703	3.579	3.210	3.202	1.573

**Table 2:** Selection of substrate reaction time (P/N)

Samples	20 min	30 min	40 min
1	2.327	4.872	5.789
2	2.434	4.308	5.153
3	2.115	4.836	5.817
4	2.318	4.79	5.371
5	2.203	4.584	5.357
Negative	1.272	1.879	2.315

### Determination of cut-off value of indirect ELISA

**Fig. 3:**
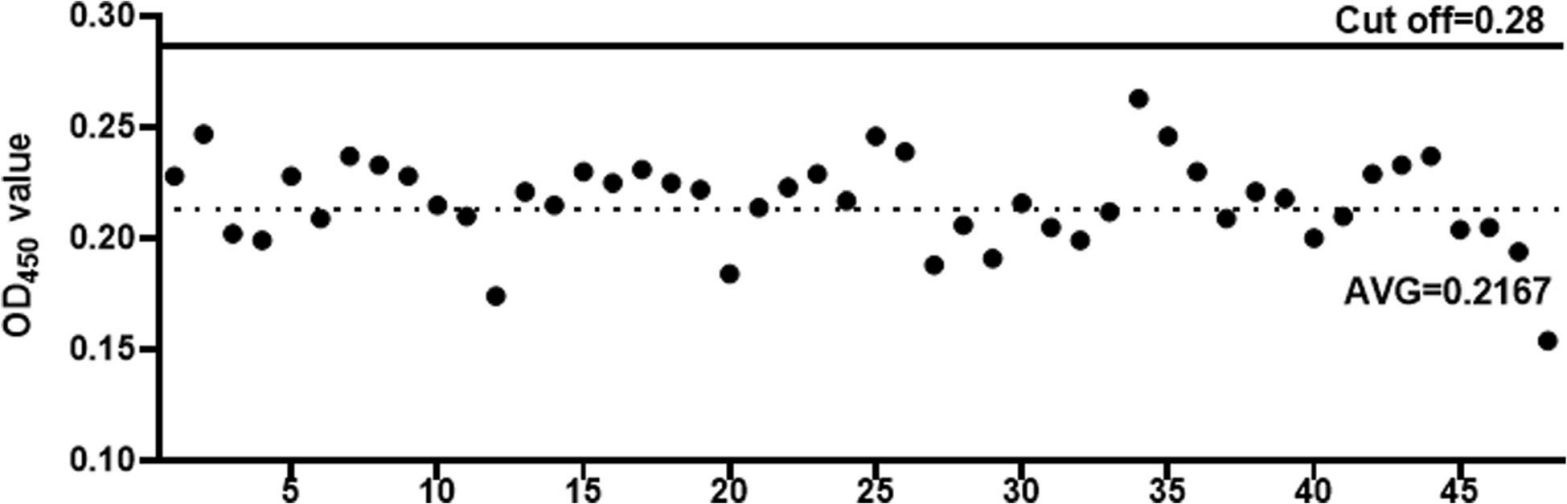
Determination of the cut-off value for the indirect ELISA. The average value plus the standard deviation of 3 times was used as the cut-off value, which was found to be 0.28. That is, if the serum OD value of the test is ≥0.28, it is judged to be positive for *Klebsiella pneumoniae* antibody; OD values < 0.28 are judged to be negative

To determine cut-off value of positive and negative samples, forty-eight goat negative serum samples without *Klebsiella pneumoniae* antibodies were detected by the indirect ELISA method. The results showed that the average value of OD_450_ values was 0.2167 and the standard deviation was 0.0198. According to the previously reported formula, the cut-off value was determined to be 0.28 (Fig. 3). Therefore, we could determine that the goat serum samples are positive if the OD_450_ value is 0.28 or above; otherwise, the samples could be determined to be negative.

### Sensitivity, specificity, and reproducibility analysis of indirect ELISA

As shown in Table 3, the P/N value was above 2.1 at a dilution of 1:800 of positive serum, while the corresponding P/N value was below 2.1 at a dilution of 1:1600. Therefore, the analytical sensitivity of the indirect ELISA method was 1: 800 dilution of serum to be tested.

**Table 3:** Indirect ELISA sensitivity measurement

	OD_450_ values of different dilutions of the *Klebsiella pneumoniae* positive and negative serum				
Bacterial dilution	1:100	1:200	1:400	1:800	1:1600
1:800	0.892 (+)	0.758 (+)	0.324 (+)	0.293 (+)	0.241 (−)
1:800	0.162 (−)	0.163 (−)	0.152 (−)	0.126 (−)	0.146 (−)

Note: (+) means positive serum, (−) means negative serum

**Fig. 4:**
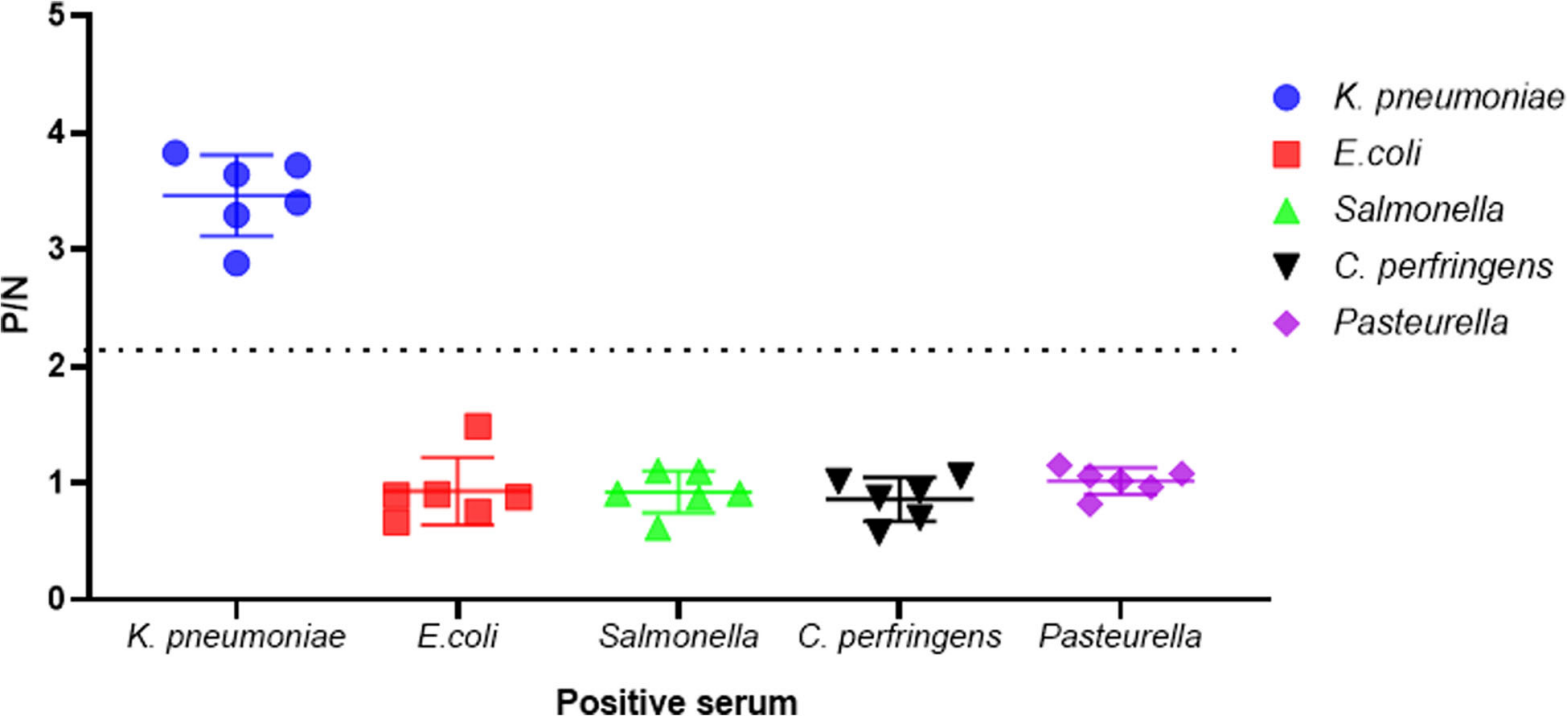
Specificity analysis of the indirect ELISA. Positive goat serum for common pathogens, including *Klebsiella pneumoniae, Escherichia coli*, *Salmonella*, *Clostridium perfringens*, and *Pasteurella* were detected by the indirect ELISA and the P/N values were recorded. The P/N values of the other four anti-serums were below 2.1 except that of *Klebsiella pneumoniae*

To investigate the specificity of the indirect ELISA, goat sera that were positive of *Escherichia coli*, *Salmonella*, *Clostridium perfringens*, and *Pasteurella* were used as the samples and followed by the established procedures. As shown in Fig. 4, the P/N values of positive reactions of *Klebsiella pneumoniae* positive serum were above 2.1, while that of negative reactions were below the value. The results indicated that the *Klebsiella pneumoniae* antigen did not cross-react with other sera, indicating that the established indirect ELISA had a strong specificity.

The reproducibility of the assay was evaluated by determining the average intra-assay and inter-assay CVs. The intra-assay CVs ranged from 1.78 to 7.20%, with a mean of 4.37% (Table 4), while the inter-assay CVs ranged from 4.06 to 6.63%, with a mean of 5.17% (Table 5). The CVs of all samples was no more than 10%, indicating that the method had good stability and reproducibility.

**Table 4:** Intra-assay of indirect ELISA

Serum number	ELISA plate batch (OD_450_ values)						Average (X)	Standard deviation (SD)	Coefficient of variation (CV%)
	A1	A2	A3	A4	A5	A6			
P1	1.196	1.249	1.361	1.296	1.362	1.321	1.298	0.0597	4.60
P2	1.122	1.227	1.241	1.254	1.177	1.255	1.213	0.0483	3.98
P3	1.205	1.202	1.281	1.216	1.259	1.164	1.221	0.0386	3.16
P4	1.099	1.141	1.124	1.079	1.113	1.101	1.110	0.0197	1.78
N1	0.177	0.186	0.16	0.164	0.184	0.174	0.174	0.0096	5.49
N2	0.189	0.19	0.175	0.154	0.183	0.191	0.180	0.0130	7.20
N3	0.192	0.199	0.174	0.195	0.182	0.186	0.188	0.0084	4.46
N4	0.183	0.168	0.174	0.188	0.174	0.188	0.179	0.0076	4.26

Note: P1-P4 are positive serum samples for *Klebsiella pneumoniae*, N1-N4 are negative serum samples, and A1-A6 are samples from the same batch

**Table 5:** Inter-assay of indirect ELISA

Serum number	ELISA plate batch (OD_450_ values)						Average (X)	Standard deviation (SD)	Coefficient of variation (CV%)
	A2	B2	C2	D2	E2	F2			
P5	0.899	0.905	0.912	0.988	0.88	0.825	0.902	0.0482	5.34
P6	0.997	0.892	0.897	0.968	0.993	0.909	0.943	0.0446	4.73
P7	0.89	0.824	0.889	0.871	0.958	0.931	0.894	0.0427	4.78
P8	0.871	0.853	0.805	0.804	0.914	0.914	0.860	0.0450	5.23
N5	0.232	0.228	0.244	0.228	0.214	0.238	0.231	0.0094	4.06
N6	0.126	0.118	0.135	0.119	0.134	0.113	0.124	0.0082	6.63
N7	0.224	0.217	0.213	0.221	0.211	0.185	0.212	0.0128	6.04
N8	0.167	0.164	0.186	0.183	0.173	0.176	0.175	0.0079	4.52

Note: P1-P4 are positive serum samples for *Klebsiella pneumoniae*, N1-N4 are negative serum samples, and A2-F2 are samples from 6 different batches

### Clinical veterinary application of indirect ELISA

A total of 1320 goat sera samples were tested for *Klebsiella pneumoniae* by the indirect ELISA and agglutination assay simultaneously. Our results showed that the positive samples detected by agglutination assay were also determined to be positive by the indirect ELISA. Moreover, the number of positive samples detected by indirect ELISA is more than that of agglutination assay, and the positive detection rate of indirect ELISA is 6.74% higher than that of the agglutination assay. These results suggested that compared with agglutination assay, the indirect ELISA test has higher sensitivity and can be applied for clinical veterinary detection of *Klebsiella pneumoniae* in goat husbandry (Table 6). Furthermore, to avoid false positives of indirect ELISA, the goats determined to be *Klebsiella pneumoniae* positive by using indirect ELISA but negative according to agglutination assay were tracked to the goat farms according to the Sample IDs and examine their physical conditions, including temperature, mental state, and whole blood cell analysis. The percentage of ELISA positive animals showing clinical symptoms was 80.9% (positive rate 72/89).

**Table 6:** Clinical veterinary investigation of antibodies against *Klebsiella pneumoniae* using the indirect ELISA and agglutination test

Serum source	Total	Indirect ELISA		Agglutination test		Co-positive	Negative samples	Coincidence rate
		Positive samples	Positive rate	Positive number	Positive rate			
Jinan	60	6	10.00%	1	1.67%	1	53	16.67%
Zaozhuang	120	3	2.50%	0	0.00%	0	117	0.00%
Qingdao	60	9	15.00%	1	1.67%	1	50	11.11%
Linyi	60	1	1.67%	0	0.00%	0	59	0.00%
Zibo	60	4	6.67%	0	0.00%	0	56	0.00%
Liaocheng	120	12	10.00%	2	1.67%	2	106	16.67%
Yantai	60	1	1.67%	0	0.00%	0	59	0.00%
Heze	60	8	13.33%	3	5.00%	3	49	37.50%
Jining	120	3	2.50%	1	0.83%	1	116	33.33%
Sunshine	60	2	3.33%	0	0.00%	0	58	0.00%
Binzhou	60	18	30.00%	4	6.67%	4	38	22.22%
Weifang	120	7	5.83%	1	0.83%	1	112	14.29%
Dongying	120	9	7.50%	2	1.67%	2	109	22.22%
Weihai	60	4	6.67%	1	1.67%	1	55	25.00%
Texas	120	21	17.50%	4	3.33%	4	95	19.05%
Taian	60	2	3.33%	1	1.67%	1	57	50.00%
Total	1320	110	8.33%	21	1.59%	21	1189	19.09%

## Discussion

*Klebsiella pneumoniae* is one of the most common Gram-negative pathogens posing a huge threat to human and animal health. In the livestock farming industry, *Klebsiella pneumoniae* could cause goat pneumonia, intracerebral hemorrhage or even death due to unsanitary living conditions. Furthermore, emerging antibiotic resistance in some strains of *Klebsiella pneumoniae* have added to this challenge. Therefore, the early detection of *Klebsiella pneumoniae* has become extremely crucial.

Currently, isolation and cultivation and agglutination assay are primary methods for the detection of *Klebsiella pneumonia* in goats [13]. ELISA is a sensitive technique based on antigen-antibody binding and signal-propagation using enzymatic reactions [14]. Compared with 16 s rRNA assay, multiple PCR techniques or real-time fluorescence quantitative PCR method established in other animals, ELISA does not require complicated equipments and is not labor-intensive [15]. Previous studies have reported an indirect ELISA for the differentiation of major *Enterobacteriaceae* from glucose non-fermenters, in the context of urinary tract and bloodstream infection [16]. This anti-gram negative bacteria (GNB) indirect ELISA can be used to differentiate *Escherichia coli* from other species of *Enterobacteriaceae*, including *Klebsiella pneumoniae*, but it cannot detect *Klebsiella pneumoniae* directly because of the absence of the corresponding antibodies [16]. In this study, we prepared a *Klebsiella pneumoniae* goat polyclonal antibody and evaluated its specificity by immunofluorescence assay. The results showed strong and specific fluorescent signals response to *Klebsiella pneumoniae* smears, demonstrating its suitability as a primary antibody for the detection of *Klebsiella pneumoniae* in an immunoassay. Subsequently, an indirect ELISA based on *Klebsiella pneumonia* was established by using the bacterial antigen as coated antigen and the *Klebsiella pneumoniae* goat polyclonal antibody as the primary antibody. The reaction conditions of indirect ELISA were optimized on the basis of P/N values [17].

Then, the method was evaluated by sensibility, specificity, reproducibility, and clinical veterinary assay efficacy. Our results show that the sensitivity of the *Klebsiella pneumoniae* indirect ELISA method was 1: 800, which was significantly higher than conventional agglutination assay. The antigenic cross-reactivity test results indicated that the indirect ELISA did not cross-react with antibodies against common bacteria and was capable of detecting anti-sera to *Klebsiella pneumoniae*. Both the intra-assay and inter assay CVs were lower than 10%, which suggested that the method has good reproducibility [18]. To further evaluate the efficacy of the method, additional tests in clinical veterinary settings are essential. We examined 1320 serum samples from goat farms in Shandong Province using the indirect ELISA and agglutination assay simultaneously. Generally, animals produce relatively low antibody level in the early stages of bacterial infection. Therefore, we could not conclude the absence of *Klebsiella pneumoniae* even if the agglutination test failed to detect antibodies in serum [19]. Even though there are several positive results present in old infections or other pathogen infections, these results are sufficient to indicate that the ELISA we have established has low false positives. The indirect ELISA exerted a higher positive rate sensitivity of *Klebsiella pneumoniae* than that of agglutination assay, which suggested that developed indirect ELISA might be a promise tool for early detection of *Klebsiella pneumonia*. Taken together, these results indicate the sensitivity, specificity and stability of our indirect ELISA method and its potential utility of detecting *Klebsiella pneumoniae* in both clinical veterinary settings and the goat-farming industry.

Since goats raised in farms (in China) do not receive vaccines protecting them against *Klebsiella pneumoniae*, we could diagnose a goat infected with *Klebsiella pneumoniae* only by detecting whether the serum contained the corresponding antibodies. The major problem is that the current methods are time-consuming and labor-intensive, and are less amenable to scaling up. Hence, the key to controlling and preventing *Klebsiella pneumoniae* infection is identifying infected goats timely, improving the intestinal microflora of goats, and reducing economic losses caused by *Klebsiella pneumoniae* infection. Although our studies have few shortcomings, including the use of the whole bacteria as coated antigen instead of representative *Klebsiella pneumoniae* surface antigens, the lack of screening for monoclonal antibodies against *Klebsiella pneumoniae*, and inevitable false positives of ELISA, which we are currently pursuing. Nevertheless, our indirect ELISA method paved the road for the early detection of *Klebsiella pneumoniae* infection in goats. In addition, our data showed that the method has the characteristics of simple, sensitive, specific, and it is suitable for large-scale clinical veterinary testing, which could be used as a serological method for *Klebsiella pneumoniae* antibodies detection in goats.

## Conclusion

Collectively, we successfully established an indirect ELISA method for detecting antibodies against *Klebsiella pneumoniae* in goats. We optimized each reaction condition, and finally demonstrated that the method has the advantages of high sensitivity, strong specificity, and excellent stability, which could satisfy requirements of production. The indirect ELISA as a rapid diagnostic kit is promising for the epidemiological investigations and clinical veterinary diagnosis of goat *Klebsiella pneumoniae*.

## Methods

### Experimental strains, animals and serum

The *Klebsiella pneumoniae* strain KpY-1 was isolated in 2018 from a goat in Shandong Province and was identified by PCR and sequencing. The titer of *Klebsiella pneumoniae* was determined to be 10^6.0^ CFU/mL by Colony-Forming Units (CFU) in Luria-Bertani (LB) agar plates. Positive goat sera for *Klebsiella pneumoniae*, *Escherichia coli*, *Salmonella Enteritidis*, *Clostridium perfringens*, and *Pasteurella multocida type B* were collected from infected goats and stored at − 80 °C in our laboratory. The negative sera were standard goat serum products we purchased randomly (C0265, SL038, and G9023). A total of 1320 serum samples were provided by the Animal Disease Prevention and Control Center of Shandong Province. All of the samples were randomly collected from different farms of 16 cities in Shandong Province.

Four one-year-old Laiwu black goats were provided by Shandong Laiwu Black Goat Seed Farm. After the experiments were completed, the goats were healthy and released.

### Preparation of the *Klebsiella pneumoniae* goat polyclonal antibody

The four Laiwu black goats were immunized with the prepared *Klebsiella pneumoniae* white oil adjuvant antigen (4.0 × 10^9^ CFU/goat) by jugular intramuscular injection for five times with immunization interval of 7 days. The serum was collected after 1 week from the last immunization and prepared for further qualification.

### Agglutination assay

The collected serum was continuously diluted with normal saline and added equivalent the inactivated *Klebsiella pneumoniae* as antigen. The mixture was adequately reacted for 5–10 min. Then, the highest dilution in which agglutination occurs is the serum titer [20]. The *Klebsiella pneumoniae* goat polyclonal antibody was feasible when the serum agglutinated and the titers were higher than 8 log2.

### Indirect immunofluorescence

Inactivated *Klebsiella pneumoniae* culture (2.3 × 10^10^ CFU/mL, 20 μL) was added to a slide and fixed with 10% formaldehyde for 15 min at room temperature [21]. After rinsing the slide with PBS three times, nonfat dry milk (5%, 20 μL) was loaded and incubated at 37 °C for 30 min. Following complete rinse with PBS for three times, the *Klebsiella pneumoniae* goat polyclonal antibody (1: 200 diluted, 20 μL) that was mentioned above was loaded and incubated at 37 °C for 30 min. After the extra primary antibody was rinsed off, FITC-labeled goat anti-rabbit IgG (1: 400 diluted, 20 μL, Beyotime Biotechnology, China) was loaded and incubated at 37 °C for 30 min [22]. Finally, the slide was washed with PBS (3 times, 1 min/time) and the positive fluorescence was observed under a fluorescence microscope (Olympus BX51, China). The culture solution without *Klebsiella pneumoniae* served as control.

### Establishment of the indirect ELISA

A square titration in 96-well ELISA microplates (BIOFIL, medium binding) was implemented to optimize the conditions for detection according to a classical indirect ELISA protocol [23]. Firstly, the inactivated *Klebsiella pneumoniae* culture (2.3 × 10^10^ CFU/mL) was serially diluted from 1:100 to 1:6400. The bacteria solution (100 μL/well) were coated with different buffer (deionized water, phosphate buffer, or carbonate buffer) at 37 °C for 1 h or 4 °C for 12 h. The wells were blocked with different buffers (0.5% BSA, 1% BSA, 1.5% BSA, or 2% BSA, 250 μL/well) at 37 °C for 1 h and or 4 °C for 12 h. The *Klebsiella pneumoniae* goat polyclonal antibody was serially two-fold diluted from 1:100 to 1:102400 by using different buffers (0.5% BSA, 1.0% BSA, or 1.5% BSA). The positive and negative sera were diluted to 1:100 and served as controls (100 μL/well). Then, the plates were incubated at 37 °C for various time scale (30 min, 45 min, 60 min, or 75 min). HRP-conjugated Affinipure Rabbit Anti-Goat IgG (100 μL/well, Proteintech, USA) was optimized with different dilutions (1:1000, 1:5000, 1:10000 or 1:15000) by 1% BSA and different incubation times (30 min, 45 min, 60 min or 75 min) at 37 °C. Finally, the plates were reacted with 50 μL substrate for various time scales (20 min, 30 min, 40 min) and were measured with a microplate spectrophotometer (XMark, Bio-Rad) at wavelengths of 450 nm (OD450). The variant was single during the execution of the entire ELISA protocol and all of the samples were tested in technical triplicates. The positive value (P) was approximately 1.0, the negative value (N) was below 0.4, and thereby the maximum ratio between P and N (P/N) was no less than 2.1, which were considered to be the optimal reaction conditions [24].

### Determination of the cut-off value

The OD_450_ values of 48 goat negative serum samples were determined by the ELISA method, and the experiment was repeated 3 times. The mean and standard deviation of the OD_450_ values were calculated. The OD_450_ average value plus the standard deviation of 3 times were used as the cut-off value to determine whether a serum sample was positive or negative by the ELISA.

### Sensitivity analysis

Sensitivity analysis, also known as the lower detection limit, was estimated by end-point titration and was defined as the maximum dilution of the sample detected just above the cut-off value. Therefore, *Klebsiella pneumoniae* positive sera was serially diluted from 1: 100 to 1: 1600 and was conducted by the indirect ELISA procedures. Each dilution was tested in triplicate and the *Klebsiella pneumoniae* negative serum samples served as controls.

### Specificity analysis

The ELISA method described above was used to simultaneously detect the OD values of goat-derived sera positive for *Klebsiella pneumoniae*, *Escherichia coli*, *Salmonella*, *Clostridium perfringens*, and *Pasteurella*, verifying whether the *Klebsiella pneumoniae* goat polyclonal antibody cross-reacts with serum positive for other pathogens. The goat-derived sera without antibodies against the above mentioned pathogens served as the negative control.

### Reproducibility analysis

To evaluate the reproducibility of ELISA, intra- and inter-assays variation analyses were conducted by the indirect ELISA. Intra-assay was performed using six different ELISA plates of the same batch, inter-assay was performed using ELISA plates to detect samples from different batches. The coefficients of variation (CVs) were calculated by dividing the standard deviation of the OD value of each tested sample by its mean and multiplying the result by 100 to evaluate the reproducibility of the ELISA.

### Field sample application

The established indirect ELISA assay was applied to determine *Klebsiella pneumoniae* infection from 1320 clinical veterinary serum samples that were collected from different goat farms in Shandong province from 2018 to 2019.

## Data Availability

All data generated or analyzed during this study are included in this published article and supplementary information files.

## Abbreviations

*K. pneumoniae*: *Klebsiella pneumoniae*
ELISA: Enzyme linked immunosorbent assay
OD: Optical density
HRP: Horseradish peroxidase
IFA: Indirect immunofluorescence assay
CV: The coefficients of variation
